# Prediction of major bleeding events for patients with dual antiplatelet therapy after myocardial infarction—a validation of the PRECISE-DAPT cancer score

**DOI:** 10.1093/ehjopen/oeaf137

**Published:** 2025-12-02

**Authors:** Sacharias von Koch, Mamas A Mamas, Mohamed Dafaalla, Francesco Costa, Sasha Koul, Tomas Jernberg, David Erlinge, Moman A Mohammad

**Affiliations:** Department of Cardiology, Clinical Sciences, Lund University, Skåne University Hospital, Entrégatan 7, 222 42, Lund, Sweden; Keele Cardiovascular Research Group, Centre for Prognosis Research, Keele University, Keele Rd, Stoke-on-Trent ST5 5BG, UK; National Institute for Health and Care Research (NIHR) Birmingham Biomedical Research Centre, Institute of Translational Medicine, Birmingham B15 2TQ, Birmingham, UK; Keele Cardiovascular Research Group, Centre for Prognosis Research, Keele University, Keele Rd, Stoke-on-Trent ST5 5BG, UK; Department of Biomedical and Dental Sciences and Morphological and Functional Imaging, University of Messina, Piazza Pugliatti 1, Messina 98100, Italy; Department of Cardiology, Clinical Sciences, Lund University, Skåne University Hospital, Entrégatan 7, 222 42, Lund, Sweden; Department of Clinical Sciences, Danderyd Hospital, Karolinska Institutet, Nobels vag 6, 171 77, Stockholm, Sweden; Department of Cardiology, Clinical Sciences, Lund University, Skåne University Hospital, Entrégatan 7, 222 42, Lund, Sweden; Department of Cardiology, Clinical Sciences, Lund University, Skåne University Hospital, Entrégatan 7, 222 42, Lund, Sweden

**Keywords:** Dual antiplatelet therapy, Myocardial infarction, Bleeding risk

The simplified original PRECISE-DAPT score was developed to identify patients at high bleeding risk (HBR) by using the following variables: age, haemoglobin, creatinine clearance and prior haemorrhage.^1^ A recent article in the *European Heart Journal* by Dafaalla *et al*. found that including cancer as a binary variable in the PRECISE-DAPT improve its discrimination ability.^2^ However, the score has not been validated using an external validation cohort. The objective of this study was to validate the PRECISE-DAPT cancer score using the Swedish Registry of Information and Knowledge about Swedish Heart Intensive Care Admissions (RIKS-HIA). RIKS-HIA contains data on all patient treated at a cardiac intensive care unit in Sweden and collects extensive information on e.g. patient characteristics, medications, and procedural characteristics. With deterministic linkage using personal identification numbers, RIKS-HIA was merged with the Swedish national patient registry to collect data on cancer status. The data underlying this article cannot be shared publicly due to patients’ privacy laws. The data were provided by the Uppsala Clinical Research Center and, upon a reasonable request, other researchers can access data through this institution. For this study, RIKS-HIA was used to identify all patients discharged with dual antiplatelet therapy (DAPT) after presenting with NSTEMI or STEMI between 30th June 2010 and 30th June 2020. Patients with missing values in the variables used to calculate the PRECISE-DAPT cancer score were excluded (*n* = 6658, 5.5%), as were patient with unknown admission time (*n* = 57, <0.1%). We calculated PRECISE-DAPT score and PRECISE-DAPT cancer score, respectively, for each patient. By using these scores, patients were categorized as very low bleeding risk (score <10), low bleeding risk (score 10–16), moderate bleeding risk (score 17–25), and HBR (score ≥25). The primary outcome was major bleeding at 1 year including bleeding events leading to death or hospitalization. Major bleeding was defined using the same ICD codes as Dafaalla *et al*.^2^ and included bleeding equivalent to bleeding ARC types 2, 3, and 5. Outcomes were ascertained up until 30th of January 2021, with complete 1 year follow-up for all patients. The scores were tested using Harrells’s *C*-statistics and Somer’s *D* and are presented along with sensitivity and specificity. In addition, net reclassification index (NRI) was calculated comparing patients categorized as HBR vs. not HBR between each score.

A total of 114 095 patients were eligible, including 4304 patients with cancer. Patients with cancer were older (mean age 75 vs. 70 years), had a larger proportion of men (72.1 vs. 68.1%), and presented more often with NSTEMI (68.4 vs. 61.9%), all *P* < 0.001. Cancer patients had a higher proportion of comorbidities, including diabetes mellitus (28.2 vs. 22.3%), hypertension (60.2 vs. 32.1%), prior myocardial infarction (22.6 vs. 15.3%), prior stroke (14.2 vs. 8.7%), and prior haemorrhage (10.8 vs. 4.8%), all *P* < 0.001. For patients with and without cancer, the median haemoglobin level was 130 (IQR: 117–143) and 140 (IQR: 129–151) and the median creatinine clearance was 62.5 (IQR: 46.0–83.0) and 69.4 (IQR: 55.0–90.2), all *P* < 0.001. Patients with cancer had a larger proportion of patients treated with clopidogrel (48.4 vs. 38.4%) and a lower proportion of patients undergoing PCI (71.6 vs. 80.2%), all *P* < 0.001. The 1-year bleeding risk for patients with cancer was 5.0% compared to 2.4% for no cancer.

The PRECISE-DAPT cancer score was associated with a Harrells’s *C* of 0.66 and Somers’ *D* of 0.32, compared to 0.65 and 0.29 for the original PRECISE-DAPT score (*Figure 1*). Using the PRECISE-DAPT cancer score and the original PRECISE-DAPT score, 21.3 vs. 20.3% were classified very low risk, 19.1 vs. 21.3% low risk, 24.6 vs. 27.0% moderate risk, and 35.0 vs. 31.5% HBR. Among cancer patients, 97.0% were classified as HBR using the PRECISE-DAPT cancer score compared to 51.3% using original PRECISE-DAPT. When using the PRECISE-DAPT cancer score, the 1-year bleeding risk was 1.0% for very low risk, 1.7% for low risk, 2.4% for moderate risk, and 4.0% for HBR. The corresponding proportions for the original PRECISE-DAPT score were 1.1, 1.9, 2.3, and 4.1%. For HBR vs. non-HBR, the sensitivity was 55.7% and specificity 65.6% for major bleeding events using the PRECISE-DAPT cancer score compared to 50.8 and 69.1% using the original score. When comparing the scores, the NRI event was 0.049 and the NRI no event was −0.035, with a total NRI of 0.014, favouring the PRECISE-DAPT cancer score. In a subgroup analysis including only patients undergoing PCI, the results were consistent with the main analysis with a greater Harrells’s *C*-statistics 0.66 vs. 0.64 and Somers’ *D* 0.32 vs. 0.29 when using the PRECISE-DAPT cancer score.

**Figure 1:**
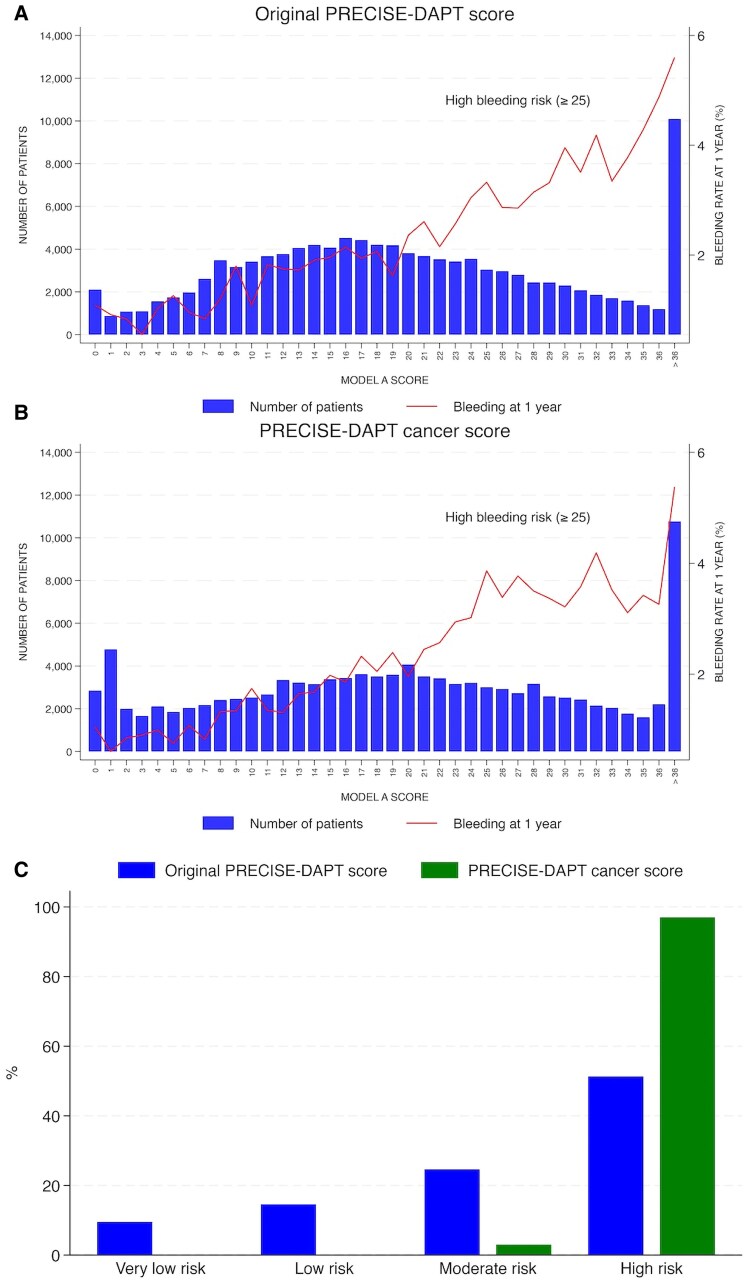
Decision curves for (*A*) the original PRECISE-DAPT score and (*B*) the PRECISE-DAPT cancer score, (*C*) proportion of patients in different risk groups in those with cancer according to the original and the original PRECISE-DAPT score and the PRECISE-DAPT cancer score.

In this nationwide observational study with the objective to validate the PRECISE-DAPT cancer score, the results confirm an improvement in bleeding prediction compared to the original PRECISE-DAPT score. Although associated with an improved discrimination of bleeding events, the clinical implication appears small with a total NRI of 0.014 and the discriminatory performance remained modest with a Harrells’s *C* of 0.66. Thus, the need to refine scores in the oncology population remains. This study presents several limitations; most important no distinction was made between active and non-active cancers, and type of cancer was not available. Instead, cancer was defined using a simplified classification that included both active and non-active malignancies. There are reasons to believe that different types of malignancies possess different bleeding risks and, consequently, the prediction of bleeding events using the PRECISE-DAPT cancer score may vary across different malignancies.

In conclusion, predicting bleeding risk in patients with acute coronary syndrome remains challenging. The PRECISE-DAPT cancer score categorized more cancer patients as HBR but resulted only in marginal improvement in discrimination of bleeding events when compared to the original PRECISE-DAPT score.

## Lead author biography

Dr. Sacharias von Koch is a research fellow at Lund University, Sweden, with a primary focus on interventional cardiology. His research involves the use of nationwide registry data to evaluate procedural strategies and clinical outcomes. Dr. von Koch is active in international collaborations and regularly presents his work at cardiology conferences. Alongside his academic work, he currently works clinically as a junior physician.

## Data Availability

The data underlying this article cannot be shared publicly due to patient secrecy laws. Interested researchers can contact us and we may help with presenting aggregated data.
